# The effect of data sources on the measurement of open access: A comparison of Dimensions and the Web of Science

**DOI:** 10.1371/journal.pone.0265545

**Published:** 2022-03-31

**Authors:** Isabel Basson, Marc-André Simard, Zoé Aubierge Ouangré, Cassidy R. Sugimoto, Vincent Larivière

**Affiliations:** 1 École de bibliothéconomie et des sciences de l’information, Université de Montréal, Montréal, Québec, Canada; 2 DST-NRF Centre of Excellence in Scientometrics and STI Policy; and Centre for Research on Evaluation, Science and Technology, Stellenbosch University, Stellenbosch, Western Cape, South Africa; 3 School of Public Policy, Georgia Institute of Technology, Atlanta, Georgia, United States of America; Humboldt-Universität zu Berlin, GERMANY

## Abstract

With the growing number of open access (OA) mandates, the accurate measurement of OA publishing is an important policy issue. Existing studies have provided estimates of the prevalence of OA publications ranging from 27.9% to 53.7%, depending on the data source and period of investigation. This paper aims at providing a comparison of the proportion of OA publishing as represented in two major bibliometric databases, Web of Science (WoS) and Dimensions, and assesses how the choice of database affects the measurement of OA across different countries. Results show that a higher proportion of publications indexed in Dimensions are OA than those indexed by WoS, and that this is particularly true for publications originating from outside North America and Europe. The paper concludes with a discussion of the cause and consequences of these differences, motivating the use of more inclusive databases when examining OA, especially for publications originating beyond North America and Europe.

## Introduction

Over the past 30 years, the democratization of the internet has made it possible for researchers, journals, and publishers to provide free online access to scholarly papers. This practice, also known as open access (OA), allows anyone with an internet connection to access, read, distribute, and download scientific publications for free with no legal or technical barriers [1]. OA publishing is no longer a marginal phenomenon, thanks to a massive rise in OA mandates [2], the introduction of several new OA publishers and OA options for legacy publishers [3], the creation of open-source software that facilitates the production of publications (such as the Public Knowledge Project), and the rise of OA mega-journals such as PLOS ONE and Scientific Reports [4].

The advantages of OA have been well-documented: increased global visibility [5], higher citation rates [6, 7], and a better use of taxpayers’ money [8]. Several studies have attempted to assess the overall share of OA publications in the scientific literature, with results ranging from 27.9% to 53.7%, depending on the data source and period of investigation [6, 7, 9, 10]. The range of these proportions demonstrate the uncertainty and variability in these numbers. This study aims at providing a comparison of the proportion of OA as represented in two prominent bibliometric databases, Web of Science (WoS) and Dimensions, and assess how the different coverage of these two databases may affect the measurement of OA across different countries.

### Data sources

The Science Citation Index (SCI) was originally developed by Eugene Garfield [11] to help librarians and researchers find articles and journals relevant for their work through citation indexing. Since it was impossible to manually index the entire range of journals (~50,000 at the time [11]), only the most cited periodicals were indexed. For decades, WoS remained the main—if not only—source of large-scale bibliometric data. However, over the past 15 years, there has been a multiplication of new data sources such as Scopus (2004), Google Scholar (2004), Microsoft Academic (2016), and more recently, Dimensions (2018). The different approaches to indexation lead to inevitable differences in coverage, which have been well-studied in several previous investigations [12–17].

For instance, Mongeon and Paul-Hus [13] have shown that, compared to Scopus, WoS has a significantly lower coverage of research in all fields, and is also much less likely to index journals from non-English-speaking countries and developing countries [13, 18]. Dimensions has much broader coverage than both WoS and Scopus [16, 19, 20]. This is largely explained by the fact that Dimensions uses Crossref (among other sources) to populate the database and focuses on a single variable for inclusion (i.e., the presence of a Digital Object Identifier (DOI)) rather than on selective criteria (e.g., citations or reputation). Despite the lack of selectivity, there are journal articles not indexed by Dimensions that are indexed by Scopus, due to the lack of a DOI across all publications [16]. However, Dimensions remains—by far—the largest and broadest indexer of scientific documents. It remains to be seen, however, whether the use of this database produces different outcomes in studies of OA.

### Country differences in OA practises

Countries differ in the proportion of their publications that are OA [6, 9, 21]. One explanation is merely one of disciplinary differences: there are well-established differences in OA practices across disciplines [6, 22] and countries differ in their disciplinary profiles [23, 24]. Policy can also drive differences, with institutional and government mandates varying in both their scope and intensity across countries [2]. These differences often intersect, in sometimes unexpected ways, with levels of economic development. For example, Iyandemye and Thomas [25] found regional differences in OA publication in biomedicine, with low-income countries and countries in sub-Saharan Africa showing a high percentage of OA publication, moderate OA publication in North America and Europe, and low participation in North Africa and South Asia. They suggested a combination of article processing charge (APC) waivers, self-archiving infrastructure, and funder policies could be contributing to these differences between countries.

The approach used by developing and developed countries for OA dissemination have historically been different [5, 10]. Developed countries tend to make use of repositories, with self-archiving mandates in place at many institutions [26] and funders [2]. These mandates may be supported by corresponding infrastructure, such as the government-funded PubMed repository or institutionally-supported repositories. Repositories are less prevalent in developing countries, as reported by the Registry of Open Access Repositories (http://roar.eprints.org/). Conversely, authors from developing countries tend to make use of OA journals [27] with various initiatives in these countries and regions which specifically focus on supporting local journals and launching OA journals to promote research from their regions. Such platforms include AJOL (Africa), AmeliCA (Latin America), and SciELO (Brazil).

In addition, OA is built on the assumption that internet access is a basic public utility that is reliably and conveniently available to everyone. This flawed assumption places developing countries at a significant disadvantage when discussing, implementing infrastructures to support, and benefitting from OA [28]. For example, in 2018, nearly 75% of the African population did not have access to the internet [29]. This lack of (affordable) internet access sometimes extends to researchers at African universities [30]. This assumption extends to the affordability of OA for researchers. APCs could make it prohibitively expensive for researchers from developing countries to render their articles OA through hybrid OA and APC charging OA journals. Full APC waivers for researchers from low-income countries, as opposed to partial waivers for middle income countries, could also be contributing to differences in OA publication practised [25, 31].

## Materials and methods

We investigated all journal-based publications indexed in WoS and Dimensions for publication years 2015 to 2019 for which first author country affiliation data was available. Both data for Dimensions and WoS were obtained from the data providers and transformed into SQL databases for data compilation. OA status for WoS papers was obtained by linking the database with Unpaywall (see, Simard et al. [10]). For Dimensions, the Unpaywall OA status of papers was already provided in the data. We used Unpaywall’s five-categories classification system [7] to discuss the OA status of publications:

- **Gold**: Published in an OA journal that is indexed by the Directory of Open Access Journals (DOAJ).
- **Green only**: Toll-access on the publisher page but is free in an OA repository.
- **Hybrid**: Free under an OA license in a toll-access journal.
- **Bronze**: Free to read on the publisher page, but without a clearly identifiable license.
- **Closed**: All other publications, including those shared only on an Academic Social Network (ASN) or in Sci-Hub.

While papers can be self-archived (green OA) and published as OA through a journal (hybrid, bronze, gold), in this study we assigned only one OA category to a paper, giving priority to journal-based OA status. Each publication was assigned to a single country based on the country affiliation that appeared on the publications for the first author. We then used the World Bank Country Classification to assign each publication into a region [32]. The different datasets were linked using the country ISO 2 alpha codes. The document types included for this study are articles and reviews for WoS and articles for Dimensions. However, the definition of “article” differs in the two indexes. While WoS classifies documents published in journals into a wide range of documents—with articles and reviews considered as peer-reviewed documents and used in measures of research production [33, 34]—, Dimensions classifies all journal documents as articles. This includes documents generally excluded from bibliometric studies—such as meeting abstracts—without the option to exclude them [16]. While this approach limits the comparability of the two datasets due to the different document types included, this is a limitation of the data sources, and reflects the most accurate representation of research production currently achievable by both indexes, thus this approach is aligned with the objective of this study. In both databases, we only considered documents with at least one institutional address. The datasets were analysed, and the figures generated, using R [35–39]. While the subject areas in which researchers and institutions are active or specialise in differ between countries and OA practises and level of engagement differ across disciplines, no field-normalisation is required for this study as the aim of the study is to compare countries with themselves between the two databases. Over the 2015 to 2019 period, WoS indexed a total of 8,053,050 publications for which affiliation data is available. Dimensions indexed 10,743,016 such publications.

## Results

**Fig 1:**
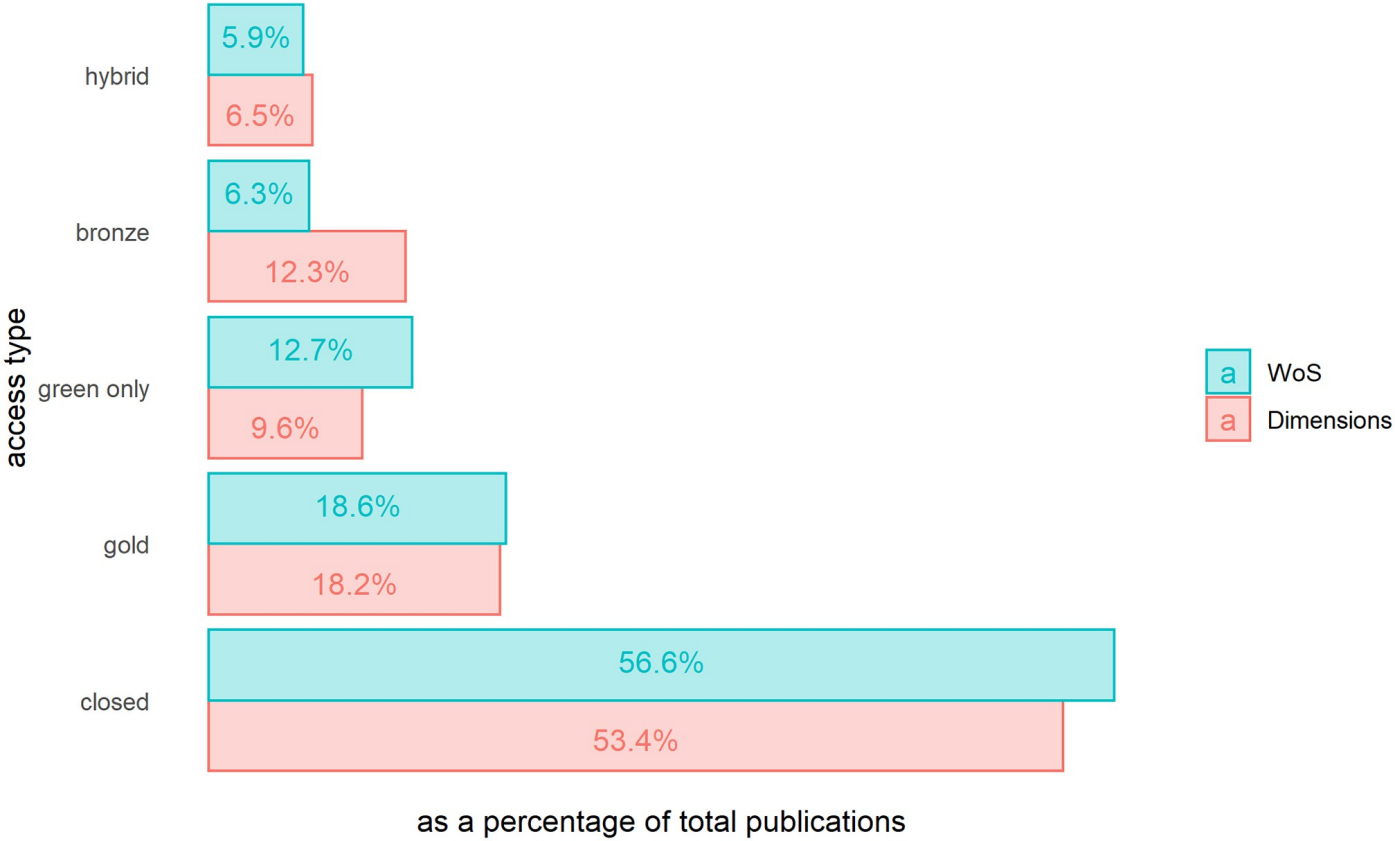
Percentage of open access publications, by access type and database, 2015–2019.

Of WoS and Dimensions publications with affiliation data, 43.4% of WoS publications and 46.6% of the Dimensions publications are available as OA publications, as shown in Fig 1. The largest differences observed between the two datasets are for the “green only”, and “bronze” categories, with a larger percentage of OA publications in WoS for the former, and a larger percentage in Dimensions for the latter.

**Fig 2:**
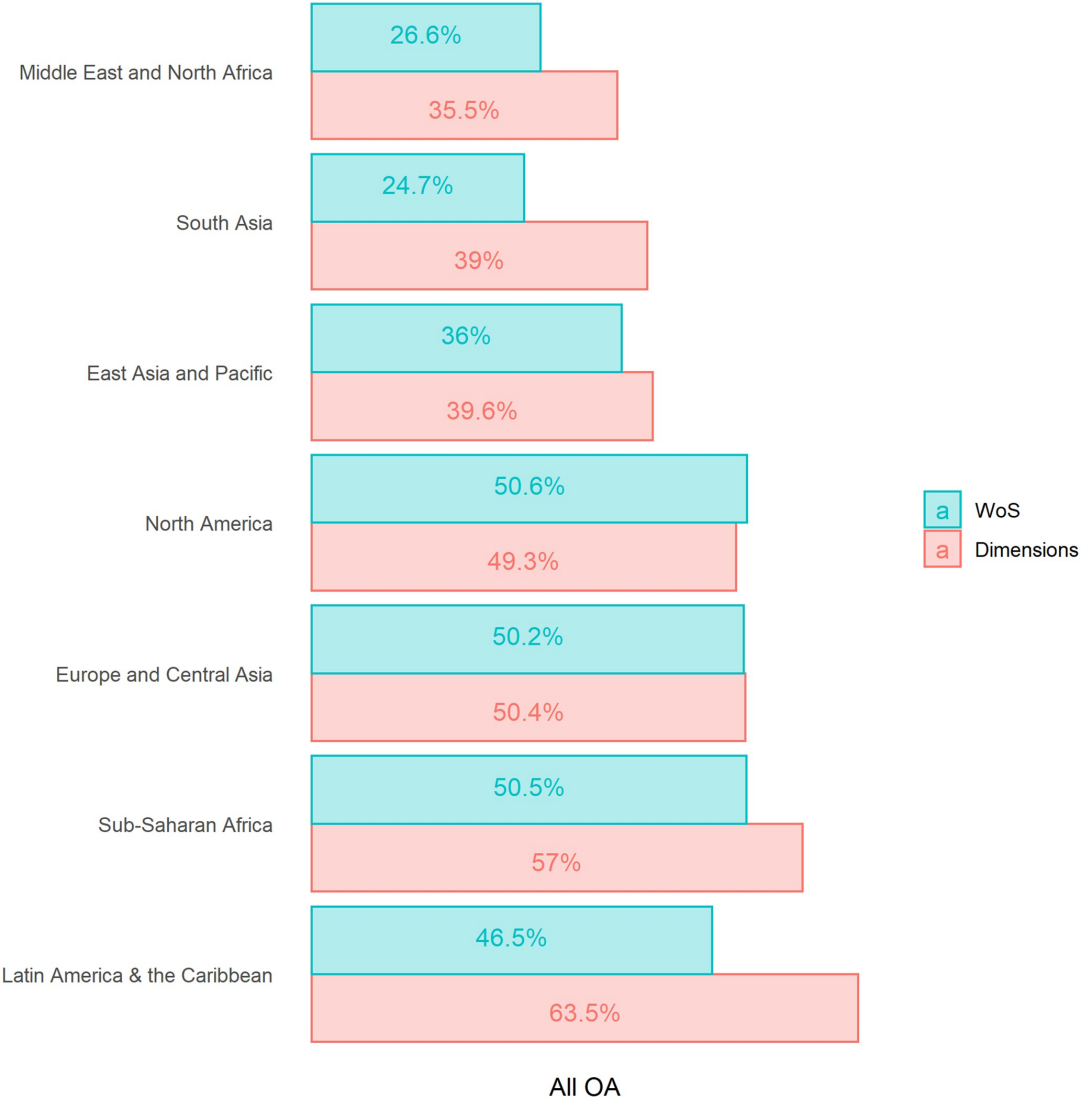
Percentage of open access publications, by region and database, 2015–2019.

Strong differences can be seen when examining according to regions (Fig 2). For developed regions (Europe & Central Asia, North America), the percentage of OA publications is similar in both WoS and Dimensions. For all the other regions—which correspond to less developed parts of the world—, the percentage of OA publications in Dimensions are significantly higher than in WoS, especially for South Asia (+57.9%), Latin Americas and the Caribbean (+36.6%), the Middle East and North Africa (+33.5%) and, to a lesser extent, Sub-Saharan Africa (+12.4%).

**Fig 3:**
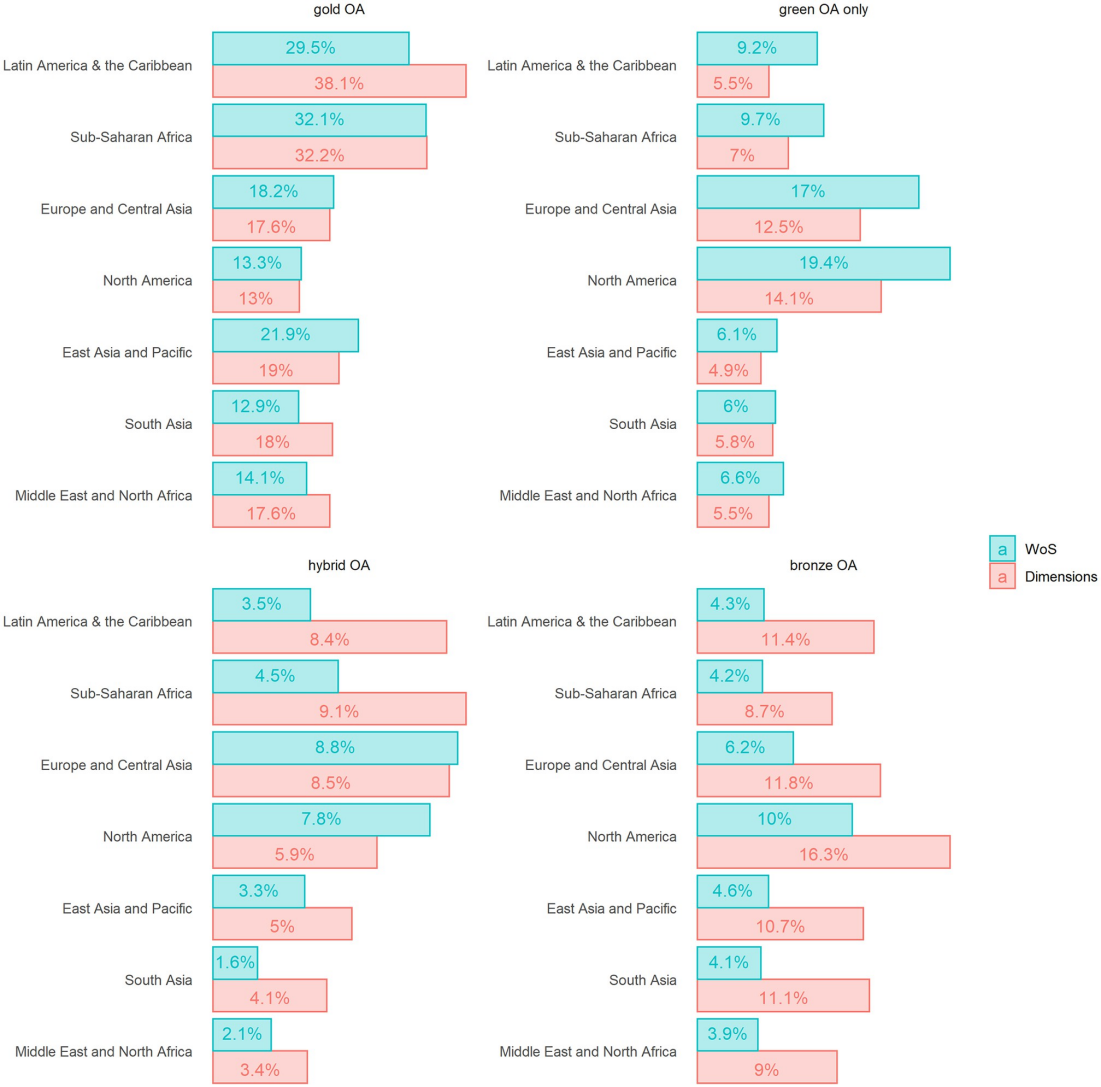
Percentage of open access publications, by region, open access type and database, 2015–2019.

These proportions differ substantially when considering different types of OA (Fig 3). For all regions but North America, the single most abundant type of OA, regardless of database, is gold OA. For North America, the most abundant type of OA is green only OA (19.4%) in WoS, and bronze OA (16.3%) in Dimensions. The percentage of gold OA is substantially higher in Dimensions than in WoS for South Asia (+28.3%), Latin Americas and the Caribbean (+22.6), and the Middle East and North Africa (19.9%) with only a slight difference for Sub-Saharan Africa (+0.3). However, it is higher in WoS for East Asia and Pacific (+13.2), with only a slight difference for Europe and Central Asia (+3.3), and North America (+2.3%). For Asia and the regions in the southern hemisphere, very few publications (<10%) are green only OA. For almost all regions, a larger percentage of publications in WoS are green only OA than in Dimensions. The exception is South Asia (+3.3), with only a slight difference in favour of Dimensions. The percentage of hybrid OA is low (<10%) for all regions, regardless of database used. The percentage of hybrid OA is higher in WoS for both North America (+24.4%) and, to a lesser extent, Europe and Central Asia (+3.4). For all other regions, the percentage is higher in Dimensions. For bronze OA, the percentage is substantially higher in Dimensions than for WoS for each of the regions.

**Fig 4:**
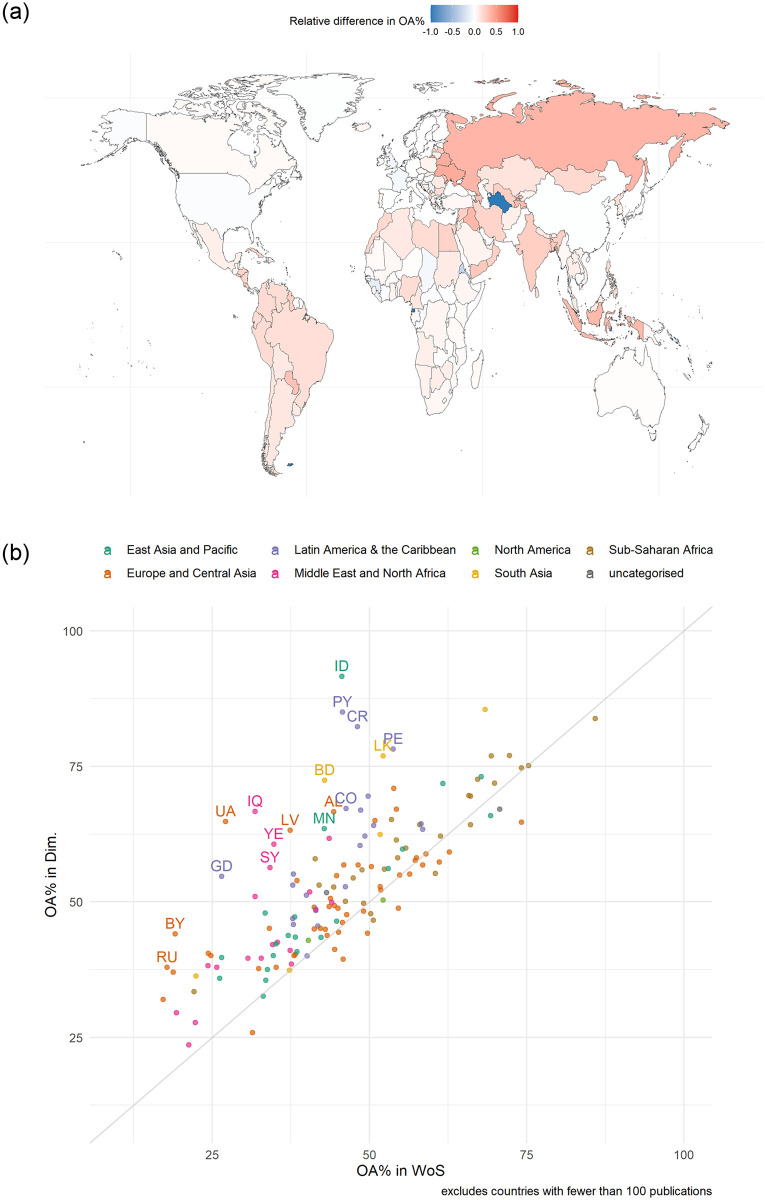
A) Relative difference in percentage of open access publications between Dimensions and WoS, by country, 2015–2019, B) Scatterplot of the percentage of open access publications for Dimensions and WoS, by country and region (includes only countries with more than 100 publications, ISO 2 code for countries with 20< percentage point difference), 2015–2019.

Basson et al. [40] found that the percentage of OA publications for some countries are significantly lower when using WoS data as opposed to when Dimensions data is used. Fig 4A and 4B illustrate this for each country by examining the relative difference in the percentage of OA publications indexed in Dimensions compared to the percentage of OA publications indexed in WoS (see [41] for data underlying for the figures). In Fig 4A, $\frac{\left(x-y\right)}{\left(x+y\right)}$, with *x* representing the percentage of papers for the country in the Dimensions dataset that are OA, and *y* representing the percentage of papers for the country in the WoS dataset that are OA, was used to calculate the relative difference between the percentages. This results in a measure where a value of -1 indicates countries for which Dimensions indexes no OA publications, whereas a value of 1 indicates those countries for which WoS indexes no OA publications, i.e., the closer to 0 the more similar the databases are in their percentage of OA publications indexed for the country in question. In Fig 4B, countries, with more than 100 publications, are plotted on a scatterplot comparing the two percentages to illustrate the number of countries that have a higher percentage or a lower percentage when using Dimensions as opposed to WoS. The figures show, for most countries, Dimensions includes a higher percentage of OA publications than for WoS (Fig 4B), and that this particularly the case for countries in the Asia and in the global South (Fig 4A).

## Discussion

Our results show that the measurement of OA differs when using WoS or Dimensions, and that the difference is more striking for authors from outside North America, Europe, and Central Asia. Given the Western bias of journals indexed in WoS [13]—which are also indexed in Dimensions [16]—the measurement of OA in these regions does not vary much in the two databases. However, for the other regions, which generally have fewer of their journals indexed in WoS, the additional publications that are indexed in Dimensions are much more likely to be OA. More specifically, as Dimensions has much broader indexing, this higher percentage of OA publications is potentially due to the inclusion of smaller national journals.

This has implications for the distribution of different types of OA, as the literature suggests that the countries generally represented in WoS are also those that tend to more often make use of self-archiving (green OA). This is observed when investigating green only OA, the only OA type that is consistently higher in WoS than in Dimensions, and substantially higher for North America, Europe, and Central Asia. The focus on self-archiving in these regions potentially explains the larger percentage of green only OA publications in WoS, as various mandates are applicable, and many repositories are available, to these authors. The higher percentage of bronze OA in Dimensions for all regions could reflect the inclusion of many non-DOAJ listed journal publications in Dimensions. It is likely that these bronze OA publications are in journals not published by the major publishers and lack the same level of standardization in metadata, resulting in difficulty classifying the publications and their inability to be indexed in DOAJ. It is also possible that due to the broad inclusion criteria of Dimensions that some predatory journals are included in this bronze OA category (potential cases were noted during a cursory investigation of the dataset) or that these bronze publications are of document types not included in the WoS dataset used for this study (e.g., editorial material that would be included in the Dimensions dataset but not the WoS dataset). Further research is required to investigate the characteristics of the additional publications included in Dimensions.

Lastly, this difference in measurement is most clearly illustrated at the level of countries. If WoS is used to measure OA for countries, the OA percentage tends to be lower for some countries in comparison to a more inclusive database such as Dimensions. Just as OA aims to provide visibility and access to research publications beyond toll-access journals, Dimensions provides a lens to investigate a broader number of publications, as opposed to only those that are considered to be the most relevant or core by Western-centric data sources such as WoS or Scopus. However, this larger coverage is not without challenges. Despite indexing more papers than WoS, Dimensions has a larger percentage of articles with missing metadata. This is particularly an issue for affiliations [20, 42], which Dimensions standardises using the GRID (Global Research Identifier Database) system alongside its use of ORCID [19]. According to Szomszor and Adams [42] many countries (especially in Africa, South America, and Central Asia) had no institutions included in GRID. The coverage has expanded since, as illustrated by the current study, which is encouraging but illustrates that while Dimensions includes publications from a larger range of journals than WoS, bibliometric studies on the level of countries are limited by the data included in GRID. The lack of affiliation data observed by Guerrero-Bote, et al., [20] in Dimensions for a large number of journal documents is only partially due to the difference in document types included in Scopus and Dimensions, as Scopus excludes, and Dimensions includes document types that do not tend to have authors. This would not explain why Scopus consistently provides more documents than Dimensions when results are aggregated by country considering Dimensions is supposed to have a wider coverage than Scopus. This potentially points to the GRID system failing to provide a country affiliation to a substantial number of eligible documents.

Along those lines, the lack of disaggregation of document types within Dimensions for journal publications not only hinders the ability to examine the characteristics of Dimensions, but also limits the possibility to conduct bibliometric studies, especially comparative ones. Considering the philosophy behind Dimensions of “combining a comprehensive coverage of the scientific literature with a flexible set of filters for making selections of the literature” [19], one hopes such disaggregation by document type will be included in the future. Given this limitation, our study cannot conclusively examine whether the differences between in OA percentages observed for the two databases is affected by the document types included in the analysis.

Despite these challenges, our analysis shows that the measurement of OA may differ significantly when one looks beyond the subset of most cited journals. Ultimately, given that Dimensions indexes journals published by the many platforms developed in the South—AJOL (Africa), AmeliCA (Latin America), and SciELO (Brazil)—it has the potential to be a more suitable platform for a more inclusive measurement of OA uptake, especially of publications by authors from outside North America, Europe, and Central Asia.
